# High-throughput imaging method for direct assessment of GM1 ganglioside levels in mammalian cells

**DOI:** 10.1016/j.dib.2016.01.027

**Published:** 2016-02-03

**Authors:** Walter Acosta, Reid Martin, David N. Radin, Carole L. Cramer

**Affiliations:** a BioStrategies LC, State University, P.O. Box 2428, AR 72467, USA; b Arkansas Biosciences Institute & Department Biological Sciences, Arkansas State University, P.O. Box 639, Jonesboro, AR 72467, USA

**Keywords:** High-throughput imaging, GM1-gangliosidosis, Acid β-galactosidase, Cholera toxin B subunit

## Abstract

GM1-gangliosidosis is an inherited autosomal recessive disorder caused by mutations in the gene *GLB1*, which encodes acid β-galactosidase (β-gal). The lack of activity in this lysosomal enzyme leads to accumulation of GM1 gangliosides (GM1) in cells. We have developed a high-content-imaging method to assess GM1 levels in fibroblasts that can be used to evaluate substrate reduction in treated GLB1^−/−^ cells [1]. This assay allows fluorescent quantification in a multi-well system which generates unbiased and statistically significant data. Fluorescently labeled Cholera Toxin B subunit (CTXB), which specifically binds to GM1 gangliosides, was used to detect *in situ* GM1 levels in a fixed monolayer of fibroblasts. This sensitive, rapid, and inexpensive method facilitates *in vitro* drug screening in a format that allows a high number of replicates using low working volumes.

## Specifications table

**Table:** 

Subject area	*Biology.*
More specific subject area	*Inborn errors of metabolism.*
Type of data	*Fluorescence microscopy images, tables, figures.*
How data was acquired	*BD Pathway 855 High Content Bioimager (BD Biosciences).*
Data format	*Raw, segmented, analyzed.*
Experimental factors	After attachment in a black wall, clear bottom 96 well plate, normal (*GLB1*^+/+^) and GM1-gangliosidosis (*GLB1*^−/−^) fibroblasts were incubated untreated or treated with recombinant β-gal for 24 h.
Experimental features	After treatment, cells were fixed, permeabilized and stained with fluorescently labeled CTXB.
Nuclei were counter stained with DAPI to allow cell count.
Individual images were acquired in each well using both filters.
Images were segmented using Attovision software.
Segmentation data were analyzed using BD Data Explorer®.
CTXB pixels per cell were calculated in each image-well.
Treatment values were expressed as average pixel/cell.
Data source location	*Images collected in Jonesboro, Arkansas, USA.*
Data accessibility	*Data is with this article.*

## Value of the data

• We describe an imaging method that statistically differentiates levels of GM1 gangliosides in mammalian cells [1].
• These data describe a sensitive, rapid, and unbiased high-throughput imaging method that allows quantification of GM1 gangliosides *in situ*.
• This method can be easily used in primary compound screening or for the testing of post-primary treatment conditions, due to advantages of the low required level of sample processing and treatment volumes.
• This assay can be adapted to multiple high-content-imaging instruments [2].

## 1 Data

High-content screening allows quantification of data obtained by fluorescence imaging in a multi-well format. Using this technology, we were able to statistically differentiate substrate levels (*p*<0.0001) between normal (GLB1^+/+^) and enzyme deficient (GLB1^−/−^) human fibroblasts. Reduction of substrate levels can be detected when GM1-gangliosidosis fibroblast (GLB1^−/−^) are treated with a corrective recombinant protein.

## 2 Experimental design, materials and methods

### 2.1 Conjugation of Dylight 594 to CTXB

For GM1 ganglioside detection, Dylight 594 (Thermo Cat #46412) was conjugated to the primary amines of Cholera Toxin B protein (CTXB) (List Biologicals Cat #104) following manufacture׳s protocol. Fluorophore conjugated CTXB has been previously used to detect GM1 gangliosides in cells (including macrophages and fibroblasts) using fluorescent microscopy [3], [4].

### 2.2 Cell treatment

Suspended Normal (GLB1^+/+^; Coriell GM-00010) and GM1-gangliosidosis (GLB1^−/−^; Coriell GM-10919) fibroblasts were diluted to 100,000 cells/ml and plated to a black-walled, clear bottom 96-well microtiter plates using 100 µl/well. Following a 24 h attachment period, media was replaced with 100 µl serum-free media (untreated) or serum-free media containing 6 nM of recombinant β-gal (R&D Systems). Cells were incubated for 24 h at 37 °C and 5% CO_2_. These parameters had been optimized to provide cell densities (impacted by cell type, cell line, growth rate, and treatment incubation times) with enough separation for accurate capture of cell count and resolution of the region of interest (ROI).

### 2.3 Fluorescence staining of cells

Cells were fixed with 4% paraformaldehyde for 8 min followed by 3X washes with PBS. Cells were permeabilized for 10 min using 0.25% Triton X-100 solution and blocked with 1% BSA+0.3 M glycine in PBS for 1 h.

Cells were incubated with conjugated CTXB-Dylight^594^ at 1:1600 dilution in PBS for 1 h at room temperature. Wells were washed 3X with PBS and cells were maintained in a PBS solution containing 600 nM DAPI and 0.03% sodium azide. Optimization of permeabilization time, Triton X-100 concentration, and CTXB-Dylight^594^ dilution was made in order to avoid signal saturation and improve assay sensitivity.

### 2.4 Image acquisition

Images were acquired with the BD Pathway 855 High Content Bioimager (BD Biosciences). A 20X NA 075 objective was used to acquire 2×2 montage images per well using DAPI (Ex=380 nm; Em=435 nm) and Texas Red (Ex=560 nm; Em=645 nm) filters. The well area acquired per each image yielded an average of 150 cells per image. All images were acquired using the same parameters summarized in Table 1 using the instrument׳s automated laser autofocus (Fig. 3).

### 2.5 Image segmentation

**Fig. 1:**
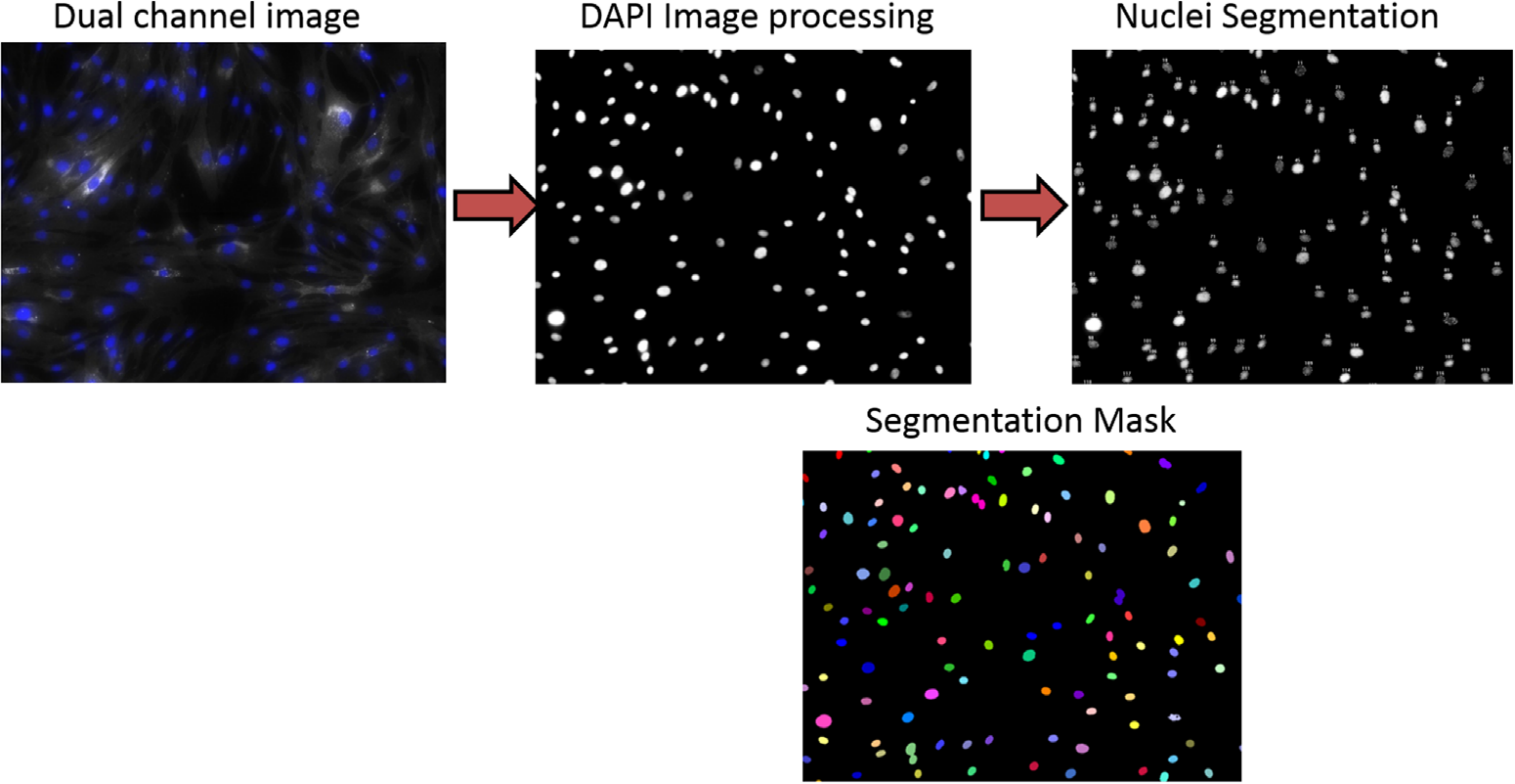
Nuclei segmentation process. After defining dye signal threshold, every image was segmented using the same parameters described in Table 2.1. Nuclei segmentation was used for cell counting.

**Fig. 2:**
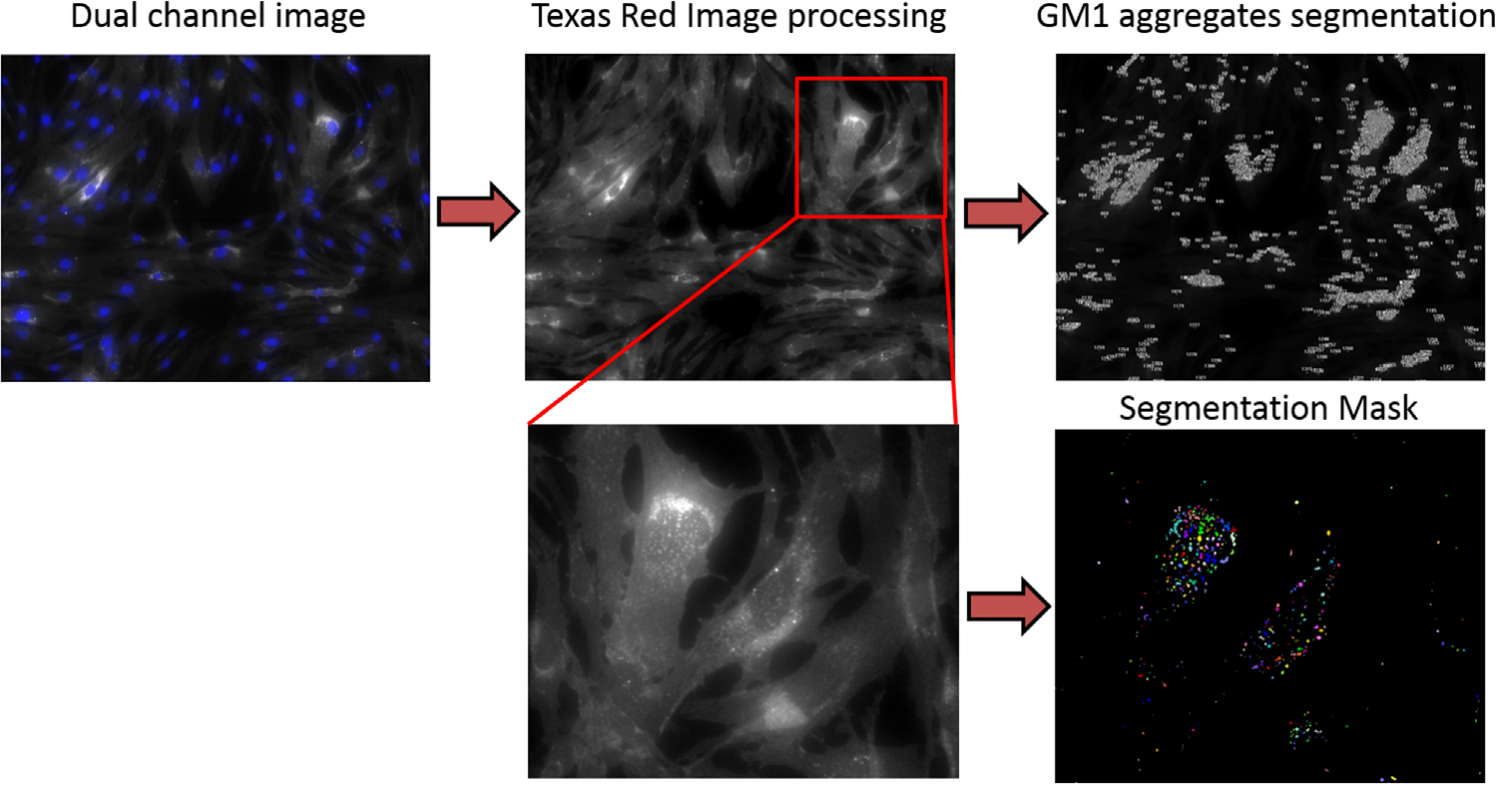
GM1 aggregates segmentation process. After defining dye signal threshold and applying filters, every image was segmented using the same parameters described in Table 2.2. Pixel area of each aggregate was used to calculate total pixels per image.

**Fig. 3:**
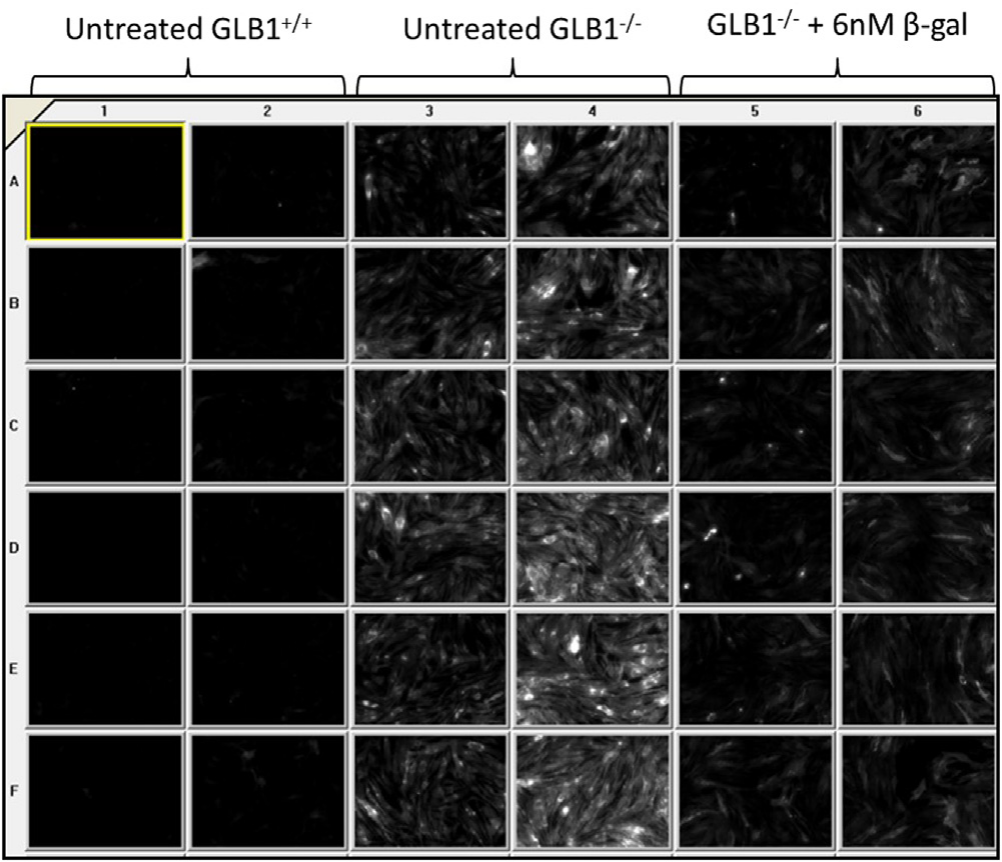
CTXB signal in different treatments. Image thumbnails of data acquired using Texas Red filter. Substrate accumulation differences are evident among treatments.

Images were segmented using Attovision software. Nuclei were counted in DAPI images by using polygon segmentation parameters described in Table 2.1. GM1 aggregates within cells were defined in Texas Red images using polygon segmentation parameters described in Table 2.2. All images were segmented using the same segmentation parameters defined for each filter channel. The segmentation process is depicted in Fig. 1, Fig. 2.

### 2.6 Data analysis

Data analysis was performed using BD Data Explorer® software (BD Biosciences). Total pixels, corresponding to GM1 gangliosides, were calculated by adding the area of one of each GM1 aggregate polygons in each image/repetition. Total pixels were divided by number of cells (nucleus count) in the corresponding image. Data were expressed as the average of CTXB-Dylight^594^ pixels per cell ratios in each treatment. Twelve images (*n*=12) per treatment were used to determine the average value (Table 3). Considering that each image included an average 150 cells at this magnification, the total amount of cells analyzed for each treatment was approximately 1800.

## Figures and Tables

**Table 1:** Acquisition parameters.

1.1. DAPI		1.2. Texas Red	
Auto dynamic range	On	Auto dynamic range	On
Dynamic range min	800	Dynamic range min	350
Dynamic range max	3200	Dynamic range max	1500
Gain	0	Gain	0
Offset	255	Offset	255
Exposure	0.3	Exposure	0.2
Lamp intensity	100	Lamp intensity	100
Excitation position A	380/10	Excitation position B	560/55
Dichroic excitation position	3 open	Dichroic excitation position	Mirror
Dichroic epifluorescence position	400DCLP	Dichroic epifluorescence position	595LP
Emission position A	435LP	Emission position A	645/75
Confocal	No	Confocal	No
Background subtraction	Off	Background subtraction	Off

**Table 2:** Segmentation parameters for nuclei and GM1 aggregates definition.

2.1. Nuclei segmentation		2.2. GM1 aggregates segmentation	
Threshold mode:	Automatic	Threshold mode:	Manual
Number of threshold steps	1	Min threshold:	294
Level 1 offset mode	Percent	Max threshold:	4095
Level 1 offset percent	0.000000	Scrap min pixels:	10
Scrap min pixels:	500	Scrap max pixels:	50,000
Scrap max pixels:	No maximum	Scrap mode:	Normal
Scrap mode:	Normal	Shape:	Polygon
Shape:	Polygon	Dilation:	0
Dilation:	0	ROI output:	Whole cell
ROI output:	Nucleus	Watershed:	Off
Watershed:	Off	Preprocessing filters:	On
Preprocessing filters:	On		
Filter2 (A)	Erode 3×3	Filter4 (A):	Top hat (7×7)
Filter3 (A)	Sharpen hat		
Filter4 (A)	RB 75×75		

**Table 3:** Segmentation quantitative data in untreated normal fibroblast, GM1-gangliosidosis fibroblast and GM1-gangliosidosis fibroblast treated with 6 nM of recombinant human β-galactosidase for 24 h.

**Untreated GLB1**^**+/+**^**fibroblasts**					**Untreated GLB1**^**−/−**^**fibroblasts**					**GLB1**^**−/−**^**fibroblasts+6 nM β-gal**				
Well ID	GM1 aggregates count	Total GM1 pixels	Cell count	Pixels/cell	Well ID	GM1 aggregates count	Total GM1 pixels	Cell count	Pixels/cell	Well ID	GM1 aggregates count	Total GM1 pixels	Cell count	Pixels/cell

A001	5	115	50	2	A003	570	10,652	94	113	A005	131	2997	99	30
A002	18	383	201	2	A004	1195	24,797	110	225	A006	649	12,237	120	102
B001	4	114	54	2	B003	2544	45,589	168	271	B005	277	5456	147	37
B002	62	975	161	6	B004	2053	43,573	124	351	B006	613	11,481	178	65
C001	6	129	84	2	C003	782	14,401	119	121	C005	117	2856	158	18
C002	48	840	122	7	C004	1616	30,804	150	205	C006	315	5520	150	37
D001	1	19	21	1	D003	1031	19,449	151	129	D005	291	6819	142	48
D002	9	197	140	1	D004	3687	67,779	186	364	D006	378	6944	193	36
E001	6	95	131	1	E003	1138	20,604	129	160	E005	386	7300	158	46
E002	26	457	115	4	E004	3746	75,338	155	486	E006	578	10,198	159	64
F001	68	1125	143	8	F003	1299	25,129	133	189	F005	421	7850	177	44
F002	16	217	119	2	F004	3356	63,919	217	295	F006	415	6854	170	40

Ave	22	389	112	3	Ave	1918	36,836	145	243	Ave	381	7209	154	47
StDev	24	382	51	2	StDev	1146	22,092	34	115	StDev	171	2952	26	22
StErr	6	102	14	1	StErr	306	5904	9	31	StErr	46	789	7	6
